
JOURNAL INFORMATION
==============================
Journal ID: Intereconomics

Intereconomics

EISSN: 1613-964X

ARTICLE INFORMATION
==============================
PMCID: PMC9362554
DOI: 10.1007/s10272-022-1069-y
Article ID: 1069
Article version: 1
Subjects: Letter from America

Inflation Fears and Strong Labor Markets

Baker Dean baker@cepr.net

grid.417588.7 0000 0004 0398 7584 Center for Economic and Policy Research (CEPR), 1611 Connecticut Ave., NW Suite 400, 20009 Washington, DC, USA
Electronic publication date: 2022 Aug 6
Print publication date: 2022
Volume: 57
Issue: 4
First page: 267
Last page: 268
Copyright: © The Author(s) 2022
License: Open Access: This article is distributed under the terms of the Creative Commons Attribution 4.0 International License (https://creativecommons.org/licenses/by/4.0/).
Open Access funding provided by ZBW — Leibniz Information Centre for Economics.
License URL: https://creativecommons.org/licenses/by/4.0/

Keywords: E31, E52
issue-copyright-statement: © The Author(s) 2022

==============================

The United States economy is in uncharted territory. On the one hand, many measures look very strong. The economy created 377,000 jobs in June 2022, bringing the total for the first half of the year to 2,740,000. The private sector has now gained back all the jobs lost in the pandemic. The unemployment rate stands at 3.6%, just 0.1 percentage point higher than its low point over the last half century. And, people are quitting jobs in numbers far higher than before the pandemic. This means that workers stuck in jobs with low pay and bad bosses now feel they have the freedom to leave.

At the same time, we see inflation hitting a 40-year high. The year-over-year rate reported in June was 9.1%. Measures of consumer confidence have sunk through the floor and many economists are now predicting a recession, while most people tell pollsters we are already in one. Also, our GDP data showed the economy shrank in the first quarter, with the data available to date indicating a decline in the second quarter is also likely. There is much to sort out here.

The first issue to deal with is the GDP data. It is absurd to imagine that the U.S. economy is currently in a recession, even if the second quarter data again show GDP is dropping. We do not have recessions when the economy is creating more than 400,000 jobs a month.

The issue here seems to be largely accounting. The negative growth in the first quarter was due to a big rise in net exports and a smaller rise in inventories than in the fourth quarter, both of which are negative entries for GDP. Final sales of domestic product, which excludes inventory accumulation and net exports, increased at a healthy 2% rate in the quarter. This is almost the same as the 1.8% growth reported for real domestic income, which in principle is just GDP added up on the income side.

GDP reportedly grew at a 6.9% rate in the fourth quarter, with inventories contributing 5.3 percentage points of this growth. It is likely that this number will be lowered in revisions and the first quarter number raised. Similarly, both consumption and investment look to be growing at a respectable pace in the second quarter, meaning that a negative number for this quarter will again be driven by inventories and/or net exports.

In short, the economy is not currently in a recession, but if the crew demanding big rate hikes from the Fed has its way, it could be soon. The claim of the recession lobby is that we are in the middle of a wage-price spiral like we saw in the 1970s. If the Fed does not raise rates aggressively, inflation will keep getting worse. Eventually, we will be faced with a choice of double-digit inflation or a deep recession, like the one engineered by Paul Volcker as Fed chair in 1981-82.

The problem with this story is that it does not fit the data. First, the labor market is hugely different in 2022 than in the 1970s. Most importantly, unions are far weaker today, with just over 6% of the private sector workforce now being unionized, compared to more than 20% in the 1970s.

We also have a far more internationalized economy. This limits the ability of domestic companies to raise prices in response to cost-pressures.

This is not just speculation. While the inflation rate has been rising through the first half of 2022, wage growth has actually slowed sharply. The annualized rate of wage growth at the end of 2021 was over 6.0%. In the most recent data, it was just 4.4% using three-month averages. If we just annualized the rate from June alone, it was 3.8%. This is only slightly higher than the 3.4% rate in 2019, when inflation was comfortably below the Fed's 2% target.

The wage data are erratic, so we have to view the exact numbers with some caution, but the direction of change is clear. Wage growth is slowing, not speeding up. We cannot have a wage-price spiral like in the 1970s if wage growth is getting slower, not faster. This is not a good story for real wages (more in a moment), but it means that we are not on the cusp of double-digit inflation.

We can also say that inflationary expectations do not seem to be getting embedded in the economy. The breakeven rate of inflation between inflation indexed Treasury bonds and regular Treasury bonds has been falling in recent months. It is now just over 2.3% for 10-year Treasury bonds. Similarly, consumer surveys show expectations of inflation are falling.

The wage-price spiral story clearly does not fit the data. Instead, the United States is seeing inflation driven by the same factors as the European Union and the rest of the world. Soaring prices for oil and gas, due to concerns about supply following Russia's invasion of Ukraine, is a huge part of the story. Similarly, the price of wheat and other agricultural commodities jumped after the invasion. We have also seen shortages of a wide range of products, from apparel to household furnishings and appliances, as a result of supply-chain problems caused by the COVID-19 pandemic.

However, we are finally working through many of these problems. Non-car inventories are more than 25% above their pre-pandemic level. The price of wheat has fallen back to its pre-invasion level and oil prices are now back below $100 a barrel, sending gas prices tumbling in the last month. Even used car prices, which had risen by almost 50% since the start of the pandemic, are now headed downward.

For these reasons, it is virtually certain that inflation will be much slower in the second half of 2022 and 2023 than it has been over the last year. This means that even if wages increase in a 3% to 4% range, workers will be able to see a healthy pace of real wage growth.

The big risk in this story is the actions of the Fed. The overnight interest rate directly under its control is still at a low level. Its hikes to date have been successful in heading off a bubble in the housing market and seem to have curbed inflation in rents as well.

However, if the Fed responds to pressure from inflation hawks, and continues to hike interest rates at a rapid pace, it will slow the economy and quite possibly throw us into a recession. Furthermore, if we do go into a recession, and the Republicans take control of at least one house of Congress, it could end up being a long one. The Republicans view a recession as their best hope of beating Biden in 2024 and will do nothing to try to boost the economy before the election.

For this reason, the biggest threat to the economy today comes from the recession lobby. If they can force the Fed to boost rates enough to have a recession, we could have a very difficult road ahead.

Dean Baker, Center for Economic and Policy Research, Washington DC, USA.

